# Short Chain Fatty Acids in the Colon and Peripheral Tissues: A Focus on Butyrate, Colon Cancer, Obesity and Insulin Resistance

**DOI:** 10.3390/nu9121348

**Published:** 2017-12-12

**Authors:** Sean M. McNabney, Tara M. Henagan

**Affiliations:** Department of Nutrition Science, Purdue University, West Lafayette, IN 47907, USA; smcnabne@purdue.edu

**Keywords:** butyrate, short chain fatty acids, obesity, type 2 diabetes

## Abstract

Increased dietary fiber consumption has been associated with many beneficial effects, including amelioration of obesity and insulin resistance. These effects may be due to the increased production of short chain fatty acids, including propionate, acetate and butyrate, during fermentation of the dietary fiber in the colon. Indeed, oral and dietary supplementation of butyrate alone has been shown to prevent high fat-diet induced obesity and insulin resistance. This review focuses on sources of short chain fatty acids, with emphasis on sources of butyrate, mechanisms of fiber and butyrate metabolism in the gut and its protective effects on colon cancer and the peripheral effects of butyrate supplementation in peripheral tissues in the prevention and reversal of obesity and insulin resistance.

## 1. Introduction

One of the widely recognized benefits of fiber consumption is the production of the short chain fatty acids (SCFAs) butyrate, propionate and acetate via fermentation in the colon [1,2,3]. Specifically, the four-carbon SCFA butyrate has been noted for its ability to directly affect the growth and differentiation of colonocytes and beneficial effects in preventing colonic cancers [4,5]. More recent studies have also shown beneficial effects of dietary fiber consumption and dietary butyrate supplementation in peripheral tissues. Specifically, increased fiber consumption or oral butyrate supplementation has been shown to decrease adiposity and improve insulin sensitivity [6,7,8,9,10]. Several tissues, including hepatic, adipose and skeletal muscle tissues, are known to possess cell surface receptors for SCFAs and exhibit beneficial alterations phenotype and physiology which may contribute to the anti-obesogenic and anti-diabetic effects of fermentation-induced SCFAs [1,11,12,13,14,15,16] and to oral or dietary butyrate supplementation [6,7,17,18]. Yet, little is known regarding the levels of butyrate which represent the threshold needed for beneficial responses within the peripheral tissues [14,19,20,21,22,23,24,25].

This review attempts to integrate the available literature relating butyrate to measurable health outcomes. Herein, we describe dietary sources of butyrate and the observed effects of butyrate in the gastrointestinal tract, including its trophic, chemopreventive and anti-inflammatory roles. We also review the action of butyrate in peripheral tissues with respect to its ability to prevent obesity and insulin resistance which may have translatable effects for the clinical treatment and prevention of obesity and type 2 diabetes (T2D).

## 2. Dietary Sources of Butyrate

### 2.1. Milk

Bovine milk fat is a particularly rich source of butyrate, with butyrate contributing approximately 4% wt/wt [26]. The fatty acid composition of milk fat varies most prominently by season, although stage of lactation and quality of feed are also mediating factors. When incorporated into a triacylglycerol molecule butyrate is most frequently esterified at the *sn*-3 position and susceptible to cleavage by pancreatic lipase in the small intestine [27,28,29]. Butyrate is generally not esterified at the *sn*-2 position, as very few dibutyryl diacylglycerols have been verified via gas chromatography [28]. This observation suggests a nonrandom esterification of fatty acids within milk triacylglycerols that may be orchestrated by stereospecific enzymes in the bovine mammary gland [27,29,30]. The positioning of butyrate within the triacylglycerol facilitates more rapid cleavage and subsequent uptake by enterocytes because pancreatic lipase liberates free fatty acids (FFAs) from the *sn*-1 and *sn*-3 positions [31]. As a consequence, butyrate released in the small intestine exerts beneficial effects on enterocyte proliferation and physiology in porcine, bovine and murine models [32,33,34,35,36].

Human breastmilk has also been examined as a potential source of butyrate for neonates and a modulator of the colonic microbiota [37,38,39]. Recent pyrosequencing experiments have identified butyrogenic bacteria in human breastmilk that may facilitate colonization of the neonatal colon [38,40]. Moreover, while human milk triacylglycerols do not contain esterified butyrate, interactions between the colonic bacteria and undigested milk metabolites may result in SCFA production [41,42]. Pourcyrous and colleagues assessed fatty acid distribution in stool samples of preterm infants fed either expressed breastmilk (EBM) or preterm infant formula (PTF) [41]. Although overall butyrate production did not differ significantly between the two conditions, the mean total SCFA concentration (μM/g stool) was significantly higher in the EBM group [41]. Interestingly, mixed model analysis revealed a diet byage interaction for butyrate [41]. Butyrate production was inversely related to postnatal age in the EBM condition, with the highest levels predicted around day 20 and a gradual decline thereafter; in the PTF group, however butyrate concentrations were lowest when full feed was initiated and the predicted concentrations increased with postnatal age [41]. Although the authors mentioned that human milk oligosaccharides may have contributed to the observed differences in the EBM group, neither oligosaccharide structure nor potential functions was analyzed from the collected samples. A similar study compared SCFA profiles in premature (gestational age ≥33 weeks) and extremely premature (<33 weeks) infants fed with fortified human milk or lactose-free formula (Nutramigen) [42]. During days 17–21, fecal butyrate concentrations were significantly higher in the formula-fed premature neonates relative to their human milk-fed counterparts [42]. Among the human milk-fed infants, those born extremely prematurely had higher butyrate percentages than those born at 33 weeks or later (22% vs. 12%) but the effect was nonsignificant [42]. Taken together, these data suggest that the infant microbiome is particularly susceptible to dietary factors that may either encourage or restrict the production of butyrate, among other SCFAs [38,40,41,42].

### 2.2. Dietary Fiber

Although dietary fiber does not contain butyrate, its fermentation by microbes in the cecum and distal colon generates SCFAs, including butyrate, which can be utilized by the host organism [43,44]. This outcome has been most consistently observed for resistant starches, complexes of amylose and/or amylopectin that have the potential to escape digestion in the small intestine [45]. Resistant starches naturally occur in foods such as cooked and cooled potatoes, raw bananas, legumes and partly milled seeds [45]. They can also be incorporated into breakfast cereals, tortillas, breads and corn (maize) through manufacturing techniques as well as fortification [45,46]. Not only do resistant starches facilitate SCFA production by the gastrointestinal microbiota [47] but they also provide systemic benefits, such as improved insulin sensitivity, when incorporated into controlled feeding trials [48,49]. For example, Gower and colleagues assessed insulin sensitivity in healthy, sedentary women following a 4-week dietary intervention with snacks containing either resistant starch (derived from high-amylose maize) in 15 g or 30 g quantities per day, or rapidly digestible starch (waxy corn) [49]. The higher dose of resistant starch significantly improved insulin sensitivity in the insulin-resistant group but it did not affect those parameters in women classified as insulin sensitive during baseline assessments [49]. It is important to note, however, that total dietary fiber intake was consistently lower in the insulin resistant group during all phases of the study relative to the insulin sensitive group and these preexisting nutritional differences may at least partially account for the experimental outcomes. A similar study examined SCFA production in healthy adults consuming test meals containing 20–25 g/day of either soluble corn fiber or resistant starch (high-amylose maize) for one week [50]. Although the soluble corn fiber treatment enhanced total SCFA production, the resistant starch treatment resulted in a greater proportion of colonic-derived butyrate [50]. These data indicate that the structural and chemical properties of dietary fibers are related to their metabolism by the gastrointestinal microbiota and particular feeding paradigms have the potential to alter microbial diversity and SCFA production in the colon [50,51,52].

The digestibility of food starches depends not only on chain length and extent of branching but also upon method of cooking, chemical pretreatments (e.g., esterification) and cooling [46]. For example, cooking at high temperatures can disrupt hydrogen bonds between amylopectin branches, thereby gelatinizing the biopolymer [45]; if allowed to cool, however, these molecules can recrystallize (retrogradation) and become more resistant to digestion by host enzymes [45,46,53]. Manufacturing processes have also been developed to decrease starch digestion in the small intestine, such as entrapping starch within calcium alginate microspheres [54]. Since these starch-entrapped microspheres ferment more slowly in the colon, even compared to other classes of resistant starches, they are less likely to induce bloating and excessive flatulence which often accompany fiber-rich diets [54,55]. Moreover, in vitro fecal fermentation assays indicated that starch-entrapped microspheres generated more butyrate during late-stage fermentation (24–48 h following inoculation) than inulin, psyllium and corn bran arabinoxylans, respectively; while total butyrate production was higher after treatment with short-chain fructooligosaccharides, long-chain β-glucan and the resistant fraction of cooked and cooled potato starch, these substrates were metabolized more rapidly and produced the majority of butyrate within 24 h of inoculation [55]. These data indicate that slowly fermenting fibers, particularly starch-entrapped microspheres, result in more reliable butyrate production in the colon over a longer time course.

The interactions between dietary fiber and the colonic microbiome are likely bidirectional. The efficiency of resistant starch fermentation is dependent on the bacterial communities present in the colon [56] and community profiles are significantly altered by both chronic feeding and single-meal interventions [44,49,50]. Unfortunately, some murine feeding studies have incorporated dietary fiber in proportions that grossly exceed recommended values for optimal human health. For example, Bindels and colleagues reported that resistant starches may comprise between 30% and 55% of total energy in rodent intervention diets [51]. While such interventions ensure that differences between the control and experimental groups are detected, their generalizability to human populations, particularly those assessed under free-living conditions, may be limited. The 2005 Adequate Intake values for total fiber were established as 38 and 25 g/day in healthy young men and women, respectively [57] but National Health and Nutrition Examination Survey (NHANES) data from 1999 to 2010 revealed an average consumption of only 16.2 g/day [58]. Unfortunately, the “Western diet” is typically rich in refined carbohydrates that contain significantly less dietary fiber [59] and so the general US population seldom consumes fiber at an optimal level.

## 3. Butyrate in the Gastrointestinal Tract

### 3.1. Effects of Microbiota on Short Chain Fatty Acid Synthesis in the Gut

Although numerous bacterial strains have been analyzed for their butyrate-producing capacities, *Faecalibacterium prausnitzii* (a member of *Clostridium* cluster IV) and *Eubacterium rectale*/*Roseburia* (*Clostridium* cluster XIVa) have currently received the most attention as they constitute 5–10% of total bacteria in fecal samples collected from healthy adults [60]. In addition to the colonization of the colon by butyrogenic bacteria, it has been proposed that cross-feeding interactions between Bifidobacterial strains and *F. prausnitzii* may ultimately enhance butyrate production [61]. When *F. prausnitzii* was co-cultured with *Bifidobacterium breve* Yakult or *Bifidobacterium adolescentis* and oligofructose (a particular inulin-type fructan) provided as the energy source butyrate was produced in appreciable quantities (between ~12 mM and ~30 mM after 48 h of fermentation) [61]. In contrast to this outcome, the effect of co-culturing *F. prausnitzii* with *Bifidobacterium angulatum* or *Bifidobacterium longum* was dependent upon the energy substrate provided; oligofructose encouraged greater butyrate production than other inulin molecules in both co-culture models, which the investigators attributed to energy competition among the bacterial strains as well as less bioavailable acetate as a co-substrate of butyrate synthesis [61]. SCFA production by the colonic microbiota has also been recognized as an important source of energy for the gastrointestinal (GI) tract cells in the host organism. For example, isolated colonocytes from germ-free C57BL/6 mice exhibited NADH/NAD^+^ ratios that were 16-fold lower than their conventionally raised counterparts, as well as 56% lower ATP levels [9]. When these germ-free colonocytes were colonized with microbes from conventionally raised mice or the butyrogenic bacterium *Butyrivibrio fibrisolvens*, their energy status improved [9]. These data indicate that the microbiome influences energy production within the GI tract.

### 3.2. Mechanisms of Butyrate Uptake and Action in the Gut

Due to the overall hydrophobicity and low molecular weights of the SCFAs in their protonated forms, acetate, propionate and butyrate can be readily absorbed via nonionic diffusion across the apical membrane of colonocytes [62,63]. Yet, the observation that <10% of SCFAs appear in the feces suggests additional mechanisms for their uptake [43]. Indeed, sodium-coupled monocarboxylate transporters (SCMTs) that utilize the colonic Na^+^ concentration gradient to efficiently sequester SCFAs within colonocytes have been identified as a mechanism of SCFA uptake [24,43,64]. Within this class of transporters, solute carrier family 5 member 8 (SLC5A8) has emerged as the primary transporter of butyrate across the apical membrane of the colonocytes and may also represent an avenue for crosstalk between the microbiome and the host organism (by exchange of metabolized products including SCFAs) [62,64]. In addition to the activity of SLC5A8, proton-coupled monocarboxylate transportation and SCFA-bicarbonate antiporters have also been proposed as viable mechanisms for SCFA uptake as well as regulators of lumen pH [23,65,66,67]. The functional overlap with regard to SCFA absorption is not surprising, as butyrate metabolism accounts for at least 70% of colonocyte energy requirements [20,62,68].

At the cell surface butyrate acts as a ligand for metabolite-sensing G-protein coupled receptors (GCPRs), including GPR43, GPR41 and GPR109A, on intestinal epithelial cells [14,69,70,71]. Due to their high affinities for SCFAs, the “orphaned” GPCRs GPR43 and GPR41 are now described as free fatty acid receptors 2 (FFAR2) and 3 (FFAR3), respectively [21,69]. FFAR2 has the potential to transduce signals through both the G_i/o_ and the G_q_ pathways, whereas GPR109A and FFAR3 only utilize the G_i/o_ pathway [21,72]. In the G_q_ family, DAG and IP_3_ may also activate protein kinase C (PKC) to ultimately stimulate the downstream activities of the extracellular signal-regulated kinase 1/2 (ERK-1/2) and c-Jun N-terminal kinase (JNK) pathways [73]. Butyrate-induced GPCR and downstream mitogen-activated protein kinase (MAPK) signaling activation that occurs through FFAR2 and FFAR3 is known to regulate inflammatory pathways that are important in determining gut health [74,75]. Activation of GPCRs by butyrate in the gut also produces the endocrine hormones glucagon-like peptide 1 (GLP-1) and peptide YY (PYY) [71,76,77]. GLP-1 is known to increase insulin secretion and FFAR2 knockout mice exhibit decreases in serum insulin levels [76]. PYY affects energy intake and expenditure at the level of the hypothalamus and brainstem and FFAR3 knockout mice exhibit blunted PYY expression [78,79,80]. Thus butyrate-induced upregulation of GLP-1 and PYY may be important in preventing or treating obesity and insulin resistance. Conversely, age-dependent increased expression of GPR109A has been noted in the jejunum of diabetic mice, where it acts to increase glucose uptake and may contribute to hyperglycemia, obesity and insulin resistance [81,82]. In addition to its GPCR second messenger and secretory-inducing functions butyrate is also a known histone deacetylase inhibitor (HDACi), targeting class I and II HDACs [83,84]. Thus butyrate may regulate epithelial cell gene expression and physiology through epigenetic mechanism involving chromatin remodeling as well as through targeting and regulation of nonhistone proteins [83]. Because the intracellular signaling effects of butyrate are pleiotropic, the physiologic consequences of butyrate are multivariate, with outcomes dependent upon tissue type, dosage and time effects. 

### 3.3. Chemopreventive Effects of Butyrate

Butyrate is known to promote growth of the colonic epithelium, yet it exerts a predominately inhibitory effect on colorectal cancers [24]). Emerging evidence suggests that the paradoxical effects of butyrate may be explained by the Warburg effect observed in various cancers [85,86,87]. Whereas noncancerous colonocytes utilize aerobic respiration to meet energy requirements, cancerous colonocytes rely upon anaerobic glycolysis even when O_2_ is plentiful [85,86]. Uptake of pyruvate by colonocyte mitochondria also decreases due to a deletion of the mitochondrial pyruvate carrier 1 (*Mpc1*) gene, an outcome that is observed among several cancers [88]. Nevertheless, the substrate-level phosphorylation that occurs during anaerobic respiration generates citrate in the mitochondrial matrix. Citrate is then exported to the cytoplasm, converted to acetyl CoA via ATP citrate lyase (ACL) and subsequently used for biosynthesis of lipids involved in cell proliferation [87]. Under conditions of anaerobic glycolysis, fatty acid oxidation is limited. Thus, the SCFA butyrate is not used extensively as an energy source by the colonocytes and begins to accumulate in the cytoplasm; this accumulation allows butyrate to act as a HDACi and ultimately sensitizes the cancerous colonocytes to apoptotic mechanisms, leading to cellular death [89,90]. Butyrate’s HDACi activity also acts to prevent macrophage-derived inflammation by downregulating production and secretion of pro-inflammatory cytokines into the gut in order to complement similar downregulation of pro-inflammatory pathways via GPCRs [91]. In noncancerous colonocytes, however, increased β-oxidation of fatty acids (including butyrate) provides a high level of acetyl CoA that can ultimately serve as acetyl group donors for histone acetyltransferase (HAT) proteins such as p300 [92]. In addition to cytosolic ACL, Wellen and colleagues demonstrated that ACL is also expressed within the nucleus [93], thereby facilitating the conversion of tricarboxylic acid (TCA) cycle-derived citrate into acetyl CoA. Nuclear acetyl CoA molecules may serve as acetyl group donors that can hyperacetylate histones [93], thereby increasing chromatin availability to the transcriptional machinery.

The ability of butyrate to accumulate in the cytoplasm of cancerous colonocytes appears to be related to the coordinated downregulation of fatty acid uptake into their mitochondria. Although it was historically established that SCFAs did not require the carnitine palmitoyltransferase proteins (CPT-1, CPT-2) for entry into the mitochondria of hepatic and cardiac tissues [94], it is important to recognize that butyrate only enters these organs in μM concentrations. In contrast, SCFAs are present in the colonic lumen at a range of 50–100 mM [95] and the CPT system appears to have a more prominent role in butyrate uptake at higher concentrations. Mechanistic analysis of the Warburg effect in HCT116 colorectal cancer cells identified decreased intracellular carnitine levels relative to noncancerous fetal human colonocytes [96]; additionally, Western blotting revealed decreased expression of organic cation/carnitine transporter 2 (OCTN2), a sodium-coupled cotransporter for carnitine, in the HCT116 cell line [96]. A similar experiment illustrated that undifferentiated Caucasian colon adenocarcinoma (Caco)-2 cancer cells exhibited negligible expression of OCTN2, whereas their mature Caco-2 counterparts (which more closely resemble small intestinal enterocytes) expressed OCTN2 at the brush border membrane [97]. Moreover, hypermethylation of the solute carrier family 5 member 8 (*Slc5a8*) gene, which codes for a sodium-dependent butyrate transporter, has been observed in both cancerous colonocytes as well as aberrant crypt foci [98]. These data suggest that impairment of butyrate uptake and metabolism characterizes early neoplasia in the colon and could potentially contribute to cancer progression [92,96,97,98]. To this end, multiple lines of research have suggested that a combination treatment of butyrate and carnitine/acetylcarnitine can exert greater effects on cancerous cells than butyrate treatment alone, possibly by enhancing butyrate localization to the mitochondria [99,100]. Oral l-carnitine supplementation also mitigated cancer cachexia symptoms in BALB/c mice injected with adenocarcinoma cells [101,102].

With the HDACi and acetylation effects of butyrate widely recognized, attention has now turned to the identification of signaling pathways through which butyrate may exert anti-proliferative and pro-apoptotic effects in cancerous tissues. For example, the transforming growth factor β (TGF-β) signaling pathway has been implicated in cell sensitization to pro-apoptotic mechanisms in noncancerous colonocytes but persistent downregulation of its downstream modulators such as mothers against decapentaplegic homolog 3 (SMAD3) has been implicated in cancer progression [103,104]. In comparison to young adult murine colonocytes, growth of Smad3^−/−^ cells was not markedly inhibited by incubation with TGF-β and ^3^H-thymidine incorporation only decreased modestly (25% decrease vs. 61% for control) [105]. MicroRNA-193b (MiRNA-193b) has also emerged as a potent inhibitor of SMAD3 and downregulation of MiRNA-193b by small interfering RNAs (siRNAs) was observed to significantly increase SMAD3 protein expression and caspase-3 activity in SW620 cells [106]. Sodium butyrate treatment in RIE-1 cells exhibited a time-dependent effect on *Smad3* mRNA content, with the longest incubation period (48 h) producing the greatest expression relative to control; moreover, sodium butyrate exhibited a dose-dependent effect on SMAD3 protein expression over a 24-h time course, with intermediate doses (2.5 and 5.0 mM concentrations) having the most potent effects [107]. Interestingly, a combination treatment of TGF-β and sodium butyrate more effectively inhibited anchorage-independent growth of RIE cells overexpressing protein kinase B (PKB/Akt) than TGF-β treatment alone [107]. Pretreatment with sodium butyrate (5 mM) followed by TGF-β treatment (40 pM) was observed to increase DNA fragmentation and the percentage of apoptotic RIE-1 cells to a greater extent than sodium butyrate alone; TGF-β treatment without butyrate did not significantly induce apoptosis or increase DNA fragmentation in comparison to the control [108]. The combination treatment also shifted the distribution of RIE-1 cells within the cell cycle, with a higher percentage of cells arrested in the G0/G1 phase and a lower percentage arrested in the S phase; interestingly butyrate treatment alone (5 mM) arrested a greater percentage of RIE-1 cells in the G2/M phase of the cell cycle, whereas TGF-β primarily arrested cells in the G0/G1 phase [108]. These data indicate that butyrate exerts unique effects on cell proliferation but it may also potentiate the effects of TGF-β signaling pathways [108]. In a similar experiment utilizing RKO, HCT-116 and HT-29 cell cultures butyrate treatment (5 mM) significantly increased the percentage of cells arrested at the G2/M phase and decreased the percentage of cells arrested in the S phase [10]. Butyrate action has also been implicated in the wingless/MMTV integration site (Wnt) pathway, likely mediated by an increased association of the cAMP-response element-binding protein (CREB) binding protein (CREBBP, or CBP) and the histone acetyltransferase p300, thereby encouraging the transcription of Wnt-related proteins involved in apoptosis of colorectal cancer cells [109,110]. Although moderate Wnt activity has been associated with cancer cell proliferation, the hyperactivation of this pathway by butyrate treatment has been demonstrated to induce apoptosis in multiple cell lines [111,112]. Unfortunately, some cancers can gradually become resistant to the effects of butyrate as well as pharmacologic HDACi [109,113]. This butyrate resistance appears to be marked by a transition from the “canonical” (β-catenin-dependent) Wnt pathway to a modified pathway that does not rely upon β-catenin for its downstream effects [113,114].

Although the tumor suppressor effects of butyrate have been attributed to its HDACi activity, it is important to note that recent studies have found that butyrate may act through the GPR109A receptor, independently of HDAC inhibition, in colon cells to prevent cancer [115]. For example, activation of GPR109A is required for IL-18 expression, leads to differentiation of regulatory T cells and anti-inflammatory IL-10 producing T cells, inhibits the pro-inflammatory nuclear factor ĸB (NF-κB) signaling pathways and causes tumor cell-specific apoptosis [115,116]. Furthermore, GPR109A deficiency increases colonic inflammation and carcinogenesis and GPR109A silencing via DNA methylation is observed in colon cancer [115,116]. Thus, the beneficial effects of butyrate on colon cancer is multi-faceted, occurring in response to both its regulation of intracellular pathways via its HDACi activity and its anti-inflammatory effects which are regulated via the extracellular GPR109A receptor.

### 3.4. Anti-Inflammatory Effects of Butyrate

Butyrate is not only responsible for the energy requirements of the colonic epithelium but it also preserves such tissues by mitigating chronic inflammatory responses through activation of its target GPCRs and its HDACi activity [9,71,74,91,115,116,117]. Both fiber-rich diets and SCFA supplementation have been associated with regulation of pro- and anti-inflammatory cytokines [69,70]. One of the most extensively studied cytokines in this regard is interleukin-8 (IL-8), which is frequently elevated in inflammatory bowel disease [118]. While IL-8 is crucial for transient recruitment of neutrophils and other cells of the innate immune system [119,120], persistent elevation of IL-8 has been reported in diabetic and sedentary individuals and it is associated with poor cardiometabolic outcomes [121,122]. IL-8 induction may also be related to macronutrient consumption. Cultured human vascular smooth muscle cells had significantly higher expression of IL-8 following treatment with palmitate, a saturated fatty acid commonly incorporated into HFDs; the effects of palmitate on IL-8 mRNA and protein content were dose dependent [123].

The effects of butyrate on IL-8 are dependent upon dose and time effects, as well as the cell type under investigation. For example, Gibson and Rosella isolated colonic crypt cells from patients diagnosed with colorectal cancer, Crohn’s disease, or ulcerative colitis and assessed IL-8 secretion in response to butyrate treatment (1 mmol/L) over a 24 h time course; in all disease groups butyrate administration significantly lowered IL-8 concentrations in comparison to control [118]. Importantly butyrate-mediated reduction in IL-8 concentration was also reported for uninflamed colonic mucosa, suggesting that the clinical utility of butyrate may extend beyond pathophysiologic conditions [118]. A similar study examined the effect of butyrate treatment on IL-8 expression following stimulation by Pam3CSK4, a pathogen-associated molecular pattern, in cultured Caco-2 and SW480 cells [124]. Concurrent treatment with butyrate significantly lowered IL-8 expression for shorter incubation periods (<9 h) but when butyrate treatment exceeded 9 h, expression of IL-8 was significantly higher than the control. Butyrate treatment also increased endogenous expression of A20 [124], a negative feedback regulator of NF-κB via ubiquitin-editing mechanisms [125,126]. In contrast to these beneficial effects butyrate incubation prior to Pam3CSK4 stimulation resulted in greater expression of IL-8 in both cell types [124], indicating that butyrate’s anti-inflammatory effects are restricted to the biochemical milieu of the tissue. Cultured HT-29 adenocarcinoma cells treated with a combination of tumor necrosis factor-α (TNF-α) and sodium butyrate exhibited reduced interleukin-8 (IL-8) secretion in comparison to cells treated with TNF-α alone [127]. The same investigators studied colonic health in Wistar rats that received butyrate enemas in tandem with a diet containing dextran sodium sulfate (DSS), an inducer of colitis in murine models; butyrate treatment resulted in statistically smaller ulcers and decreased myeloperoxidase activity relative to controls [127]. Another mechanism by which butyrate has been proposed to reduce systemic inflammation is the maintenance of the intestinal epithelial barrier [4,128]. Recent evidence has suggested that changes in tight junction localization, intestinal permeability and gut microbial diversity may precede the development of obesity and T2D [129,130]. As the mucosal layer becomes less capable of repelling unfavorable bacterial strains, these bacteria or their metabolites are able to traverse the intestinal barrier and invade the surrounding tissue, thereby stimulating an innate immune system response [129]. If poor dietary lifestyle choices, among other factors, prevent full reconstitution of the intestinal barrier, the individual may experience persistent low-grade inflammation, which has been associated with obesity and insulin resistance as well as dysfunction of the peripheral tissues [131]. In a Caco-2 cell model of the intestinal epithelium, Peng and colleagues assessed the effects of butyrate supplementation on tight junction protein expression, localization and transepithelial electrical resistance (TER) [4]. Although butyrate incubation (2 mmol/L for 72 h) did not significantly increase protein expression of claudin-1, claudin-4, zona occludens-1 (ZO-1) and occludin, the incubation increased TER and localized the tight junction proteins to the cell periphery during a calcium switch assay [4]. Moreover butyrate treatment increased the ratio of phosphorylated AMP-activated protein kinase (AMPK) to total AMPK content in a time-dependent manner. The role of AMPK as mediator of these processes was supported with the addition of compound C (10 μM), a known inhibitor of AMPK; when compound C was added to the cell system butyrate could not induce tight junction assembly even in the presence of Ca^2+^ [4]. The reparative effect of butyrate incubation was also reduced in an analogous experiment by introducing small interfering RNAs (siRNAs) to decrease expression of AMPK [5]. A similar experiment utilized SCFA mixtures with different proportions of butyrate (5%, 20% and 50%) to examine the effect of treatment on barrier function with concomitant addition of pro-inflammatory lipopolysaccharide molecules and TNF-α [128]. When the proportion of butyrate was higher in the SCFA mixture (20% or 50%), TER increased significantly despite TNF-α and lipopolysaccharide treatment; moreover butyrate incubation at the highest concentration increased TER following previous TNF-α and lipopolysaccharide treatment, suggesting that butyrate incubation can exert both protective and reparative effects on the intestinal barrier [128].

## 4. Butyrate, Obesity and T2D

Obesity and its associated pathologies represent one of the greatest emerging healthcare challenges in the developed world. In the United States alone, at least two-thirds of the adult population is classified as overweight (defined as a body mass index (BMI) ≥ 25 kg/m^2^) and greater than one-third of the adult population is obese (BMI ≥ 30 kg/m^2^) [132,133,134]. Even more troubling, the prevalence of overweight and obesity in children and adolescents assessed between 2011 and 2012 was reported as 31.8% [132]. Obesity imposes significant costs on the United States economy, as the aggregate national cost of overweight- and obesity-related medical treatment has been estimated at $113.9 billion [135]. Obesity also exerts financial strain on businesses, as greater absenteeism and workplace fatigue/dampened productivity (“presenteeism”) are associated with increasing BMI levels [136,137,138]. Moreover, obesity is associated with several pathologies including cardiovascular disease (CVD) [134,139,140,141,142], certain types of cancer [134,143,144,145,146], non-alcoholic fatty liver disease (NAFLD) [147,148], reproductive dysfunction [149,150] and T2D [151,152]. In fact, the grossly elevated risk for T2D development among overweight/obese individuals has prompted the American Diabetes Association to recommend testing of overweight adults of any age who present with one or more additional risk factors, including physical inactivity [153]. Once T2D is established, the individual is also susceptible to peripheral neuropathies, retinopathy and nephropathy [154]. Interestingly, treatments with butyrate or those that increase butyrate production, such as increased dietary fiber or bacterial colonization in the gut, have been shown to prevent or attenuate obesity and insulin resistance [6,11,12,13,17,155,156,157,158,159,160,161].

### 4.1. Butyrate and Obesity

Dietary butyrate supplementation has been shown to mitigate weight gain through attenuating increases in adiposity in animals fed high fat diets (HFD) [6,155,156]. For example, the elegant work of Gao and colleagues supported a protective effect of sodium butyrate supplementation on body weight, as C57BL/6J mice maintained on HFD and 5% wt/wt sodium butyrate gained significantly less body weight during the dietary intervention than the HFD controls [6]. Moreover, sodium butyrate-supplemented mice exhibited lower body fat percentages and higher muscle content [6]. In addition to investigating the preventative effects of butyrate on obesity, investigators also examined its ability to reverse obesity and insulin resistance following HFD feeding over a 16-week period. Butyrate was supplemented into the HFD for 5 weeks following the 16 week HFD only period and investigators observed that butyrate-supplemented mice exhibited a 10.2% decrease in body weight and 10% reduction in body fat content relative to the HFD controls [6]. These changes were also accompanied by greater insulin sensitivity, as assessed by the homeostatic model of insulin resistance (HOMA-IR) [6]. Hong and colleagues also examined the ability of oral sodium butyrate administration on reversing the deleterious effects of 8 weeks of HFD (45% kcal fat) feeding in C57BL/6 mice [18]. Investigators administered sodium butyrate (80 mg) to half of the HFD-fed mice via gavage for 10 consecutive days, while control HFD mice received vehicle. Mice receiving sodium butyrate supplementation exhibited decreased serum insulin, leptin and fasting glucose concentrations [18]. Total body weight, liver weight and epididymal fat pad weight also decreased in butyrate-supplemented mice relative to the HFD controls [18]. The protective effects of butyrate have also been observed following VSL#3 probiotic supplementation in C57J/B6 mice, with body weight and fat mass being significantly decreased after 5 weeks of VSL#3 treatment [155]. Similarly, supplementation with *C. butyricum* not only reduced body weight gain and mitigated fat pad size over a 12 week HFD intervention but it also reduced free fatty acid content in the liver, suggesting diminished ectopic hepatic lipid deposition relative to the HFD controls [156].

Importantly, the anti-obesogenic effects observed following butyrate supplementation can also be achieved through dietary interventions involving resistant starches and other fermentable fibers [11,12,13]. For example, Keenan and colleagues assessed the differential effects of low fiber (5% fiber), fermentable high amylose resistant cornstarch (39.9% fiber, 33% resistant starch) and non-fermentable methylcellulose (37.5% fiber) diets on weight and body composition in 7-month-old female Sprague-Dawley rats [11]. Rats fed the resistant cornstarch or the non-fermentable methylcellulose diets exhibited significantly lower mesenteric, gonadal and abdominal fat pad weight compared to the low fiber group [11]. Interestingly, the resistant starch diet resulted in elevated gene expression of PYY and preproglucagon in both cecal and large intestine samples; whereas, the expression of these genes in the methylcellulose diet was not different from the low-fiber control group. PYY and GLP-1 protein content was also significantly higher in the sera of resistant starch fed rats relative to the low fiber fed and methylcellulose fed rats, suggesting that resistant starch feeding can promote an anorectic effect in rodents [11]. To more thoroughly examine the importance of fiber type on these outcomes, the investigators provided 8-week-old male Sprague-Dawley rats with resistant starch or non-fermentable cellulose diets that were equivalent with regard to metabolizable energy density (3.3 kcal/g) [11]. Resistant starch fed rats exhibited lower disemboweled body weight, abdominal fat content and lower cecal pH relative to their cellulose fed counterparts. After 3 weeks of feeding, the resistant starch intervention also resulted in greater gene expression of PYY and proglucagon, as well as a significantly greater plasma concentration of PYY [11].

A similar study utilized a 2 by 2 factorial design to assess high-amylose maize resistant starch type II (HAM-RS2) and sodium butyrate dietary interventions in male Sprague-Dawley rats over the course of 12 weeks [13]. Significantly lower abdominal fat (as a percentage of disemboweled body weight) was reported for the sodium butyrate, resistant starch and combination treatments relative to the energy control group; moreover, the combination treatment exerted more prominent effects on weight management than either treatment administered individually [13]. Resistant starch treatment significantly increased serum concentrations of GLP-1 and PYY; but, the combination treatment significantly diminished this increase [13]. Sodium butyrate treatment alone did not increase serum GLP-1 significantly and it only marginally increased serum PYY levels [13]. According to the investigators, the mechanisms through which dietary butyrate and resistant starches exert their beneficial effects on weight management may differ depending upon the location of metabolism and absorption of these dietary components [13]. 

Finally, a study was conducted comparing the effects of two dietary fat levels (7% vs. 11% wt/wt) and starch compositions (amylopectin vs. resistant starch (Hi-Maize 260^®^)) on body weight and metabolic parameters in C57BL/6J mice and two polygenic murine models of obesity: NONcNZO10/LtJ and Non/ShiLtJ mice, respectively [12]. Although resistant starch treatment improved fasting glucose and HOMA-IR scores for C57BL/6J maintained on a 7% fat diet, the resistant starch did not improve these parameters in obesity-prone polygenic strains nor did it improve adiposity [12]. The investigators asserted that gut microbial diversity may differ widely among the three mouse strains and might account for the inability of obesity-prone polygenic mice to ferment resistant starches in the colon but such data were not included in the present analysis [12]. Alternatively, differences in outcomes may be due to the dietary starch type which affects production and subsequent levels of the various SCFAs in the gut. For example, in a 16-week feeding trial in C57BL/6J mice with five types of HFD, including HFDs consist supplemented with sodium acetate, sodium propionate, or sodium butyrate supplementation at 5% wt/wt, or a mixture of these three SCFAs in a 3:1:1 ratio, the sodium butyrate supplemented diet resulted in increased *Gpr41* mRNA content in the adipose tissue to a greater extent than any of the other SCFAs and to a level higher than the HFD or low-fat diet (LFD, 10% kcal) controls [162]. These data suggest that SCFA-based dietary interventions can profoundly alter gene expression in metabolically active tissues and that, under certain circumstances butyrate may exert effects that differ from those of acetate and propionate.

### 4.2. Butyrate and Insulin Resistance

In addition to its preventive effects on body weight and adiposity butyrate supplementation has also been associated with the mitigation of insulin resistance in several animal models [6,17,157,158]. For example, sodium butyrate supplementation (5% wt/wt) into the HFD (58% kcal fat) of C57BL/6J mice resulted in lower fasting glucose and insulin levels, as well as greater insulin sensitivity according to HOMA-IR [6]. Additionally, sodium butyrate-supplemented mice exhibited decreased serum triglyceride and total cholesterol levels compared to controls [6]. These data indicate that butyrate can exert beneficial metabolic effects in spite of the challenges posed by an obesogenic diet. A similar experiment assessed the effects of 1% butyrate supplementation in the drinking water of HFD fed (60% kcal fat) and low-fat diet fed (10% kcal fat) CD-1 mice and found that butyrate lowered serum insulin and fasting glucose levels compared to HFD controls [17]. Moreover, overall body weight and liver triglyceride levels were significantly lower in butyrate-supplemented HFD mice, suggesting that butyrate may prevent or otherwise reduce ectopic deposition of lipids [17]. Khan and Jena examined the effect of differential sodium butyrate injections (200 or 400 mg/kg intraperitoneal doses, twice per day) on streptozotocin-induced diabetic Sprague-Dawley rats maintained on a HFD (58% kcal fat) [157]. Following 10 consecutive weeks of treatment, rats administered the higher dose of sodium butyrate exhibited lower glycated hemoglobin (HbA1C) content, total cholesterol and plasma glucose levels relative to diabetic controls [157]. Additionally, the ratio of acetylated H3 histone content to total H3 histones was significantly elevated in both sodium butyrate treatment groups relative to the diabetic control, indicating that some of butyrate’s beneficial effects are mediated through HDAC inhibition and, by extension, greater expression of particular genes [157].

Dietary fibers that facilitate butyrate production by the gut microbiome have also been associated with greater insulin sensitivity [159,160]. For example, Zhou and colleagues maintained adult male C57BL/6J mice on a control or resistant starch rich diet for 10 days; following this period, the mice received intraperitoneal injections of vehicle (citrate buffer) or streptozotocin for 5 consecutive days while continuing their respective diets [159]. At the end of this period, an oral glucose tolerance test was performed. Area under the curve analysis indicated that streptozotocin-injected diabetic mice maintained on the resistant starch diet handled the glucose challenge significantly better than their diabetic control diet counterparts [159]. Likewise, Goldsmith et al. assessed insulin sensitivity and metabolic parameters in male Zucker diabetic fatty (ZDF) rats fed amylopectin-rich corn starch (0% resistant starch), high-amylose maize resistant starch (25% resistant starch), whole grain flour with minimal amylose content (6.9% resistant starch), or whole grain flour with 70% amylose content (25% resistant starch) [160]. All four diets were formulated to be isocaloric (3.2 kcal/g) and were well-tolerated by the rats. After 8 weeks of feeding, serum samples were collected for HOMA-IR analysis. Interestingly, only the high-amylose maize resistant starch treatment resulted in significantly lower HOMA-IR values relative to the controls [160]. Insulin sensitivity in the rats maintained on the whole grain, resistant starch-rich diet was not significantly different from the control groups [160]. Nevertheless, both resistant starch-rich diets (amylose vs. whole grain) resulted in higher serum concentration of GLP-1 and altered microbial distribution, with significant increases observed for members of the Bacteroidetes family and decreases with regard to Firmicutes members [160].

In addition to directly supplementing butyrate into the diet, multiple studies have illustrated that supplementing the diet with butyrogenic bacterial strains in the form of a well-tolerated probiotic can exert similar metabolic effects [155,156,161]. For example, C57BL/6 mice were treated with a probiotic containing the butyrogenic bacterium *Clostridium butyricum* in combination with HFD (45% kcal from fat) [156]. In comparison to the mice consuming HFD alone, mice that received *C. butyricum* probiotics in conjunction with the HFD exhibited significantly lower fasting serum insulin levels and lower blood glucose levels at both 30 and 120 min following an intraperitoneal glucose tolerance test (GTT) [156]. The investigators also reported lower total serum cholesterol, non-esterified fatty acids and LDL content for the mice that received *C. butyricum* supplementation [156]. In a similar study, C57J/B6 mice were fed either a LFD (10% kcal fat) or HFD (60% kcal fat) with or without the potent probiotic mixture VSL#3 for 8 weeks [155]. In comparison to the HFD-only condition, mice administered VSL#3 in conjunction with HFD exhibited significantly lower serum insulin, fasting blood glucose and fed blood glucose levels [155]. Additionally, VSL#3 supplementation in both the LFD and HFD groups significantly increased butyrate production as assessed by fecal sampling and the VSL#3-HFD combination treatment altered the microbiota composition, with greater DNA markers for Bacteroidetes and Bifidobacterial strains [155]. In a distinct experimental model, Li and colleagues assessed the effects of live or dead probiotic mixtures on streptozotocin-induced diabetic C57BL/6J mice [161]. Both live and dead probiotic-treated mice exhibited greater insulin sensitivity according to HOMA-IR relative to the diabetic control but only the live probiotic treatment group exhibited significantly lower insulin levels overall [161]. Moreover, HbA1C content and leptin levels were significantly lower in probiotic-supplemented mice relative to the diabetic controls [161].

These studies clearly indicate that butyrate supplementation through various means may have beneficial effects on preventing obesity and whole-body insulin resistance, which are characterized by aberrant metabolism in peripheral tissues. For example, perturbations of mitochondrial β-oxidation of fatty acids, leading to incomplete β-oxidation and ectopic lipid deposition in liver and skeletal muscle and excessive accumulation in adipose tissue is observed in the obese, insulin resistant state [163,164,165,166,167,168,169,170].

## 5. Butyrate and Peripheral Tissues

**Figure 1:**
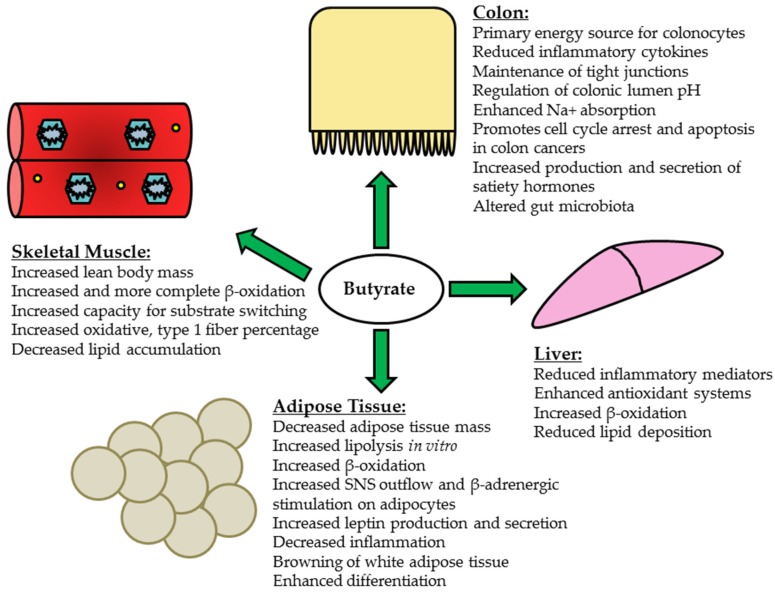
Summary of principle effects of butyrate and its potential mechanisms of action in the colon and peripheral tissues; liver, skeletal muscle and adipose tissue.

The mechanisms through which butyrate acts to attenuate and ameliorate obesity and insulin resistance are not fully understood but clearly involve contributions from various peripheral tissues, including the liver, skeletal muscle and adipose tissue (Figure 1). Yet, the delivery (or that of its metabolites) of butyrate via the systemic circulation and subsequent uptake in peripheral tissues remains largely unknown. In one of the earliest quantification experiments of human SCFA localization, Cummings and colleagues measured SCFAs in portal, hepatic and peripheral blood during autopsies of sudden death victims and found that average concentrations of butyrate were 29 μmol/L, 12 μmol/L and 4 μmol/L, respectively [171]. A more recent study assessed SCFA flux in human patients undergoing major upper abdominal surgery [22]. Unsurprisingly butyrate was very well absorbed in the gut and has been shown to be subsequently released into the portal blood at 5.7 μmol/kg body weight/h [22]. Once butyrate reaches the liver, the amount metabolized occurs in proportion to the amount released from the enterocytes and colonocytes, with the liver clearing a large portion of SCFA from the portal circulation in an effort to prevent high systemic concentrations [22]. This finding does not necessarily preclude butyrate activity in other peripheral tissues, as it has been shown that butyrate supplementation affects metabolic parameters in adipose and cardiac and skeletal muscle and various peripheral tissues possess the SCFA receptors, mainly free fatty acid receptor (Ffar) 2 and 3, needed for butyrate recognition [6,7,13,14,17,18,157]. Indeed, extensive studies showing the effects of butyrate supplementation on peripheral tissues supports a role for its direct or indirect action in the periphery.

### 5.1. Liver

Obese, diabetic models in a simplistic context exhibit fatty, insulin resistant livers due to increased triglyceride content and decreased β oxidation within the tissue, which is also associated a pro-inflammatory state. Supplementation of butyrate or butyrogenic bacterial strains is associated with the reduction of ectopic lipids in hepatic tissue [172,173,174,175]. For example, Jin et al. assessed the effect of oral sodium butyrate supplementation (0.6 g/kg body weight/day) in female C57BL/6J mice fed either a liquid control (12% energy from fat) or Westernized diet (25% energy from fat, 50% *w/w* sucrose supplementation) for 6 weeks [172]. Despite similar body weights, the mice receiving sodium butyrate supplementation in conjunction with the Westernized diet exhibited a significant reduction in intrahepatic lipid deposition, decreased liver damage as assessed by the non-alcoholic fatty liver disease activity score (NAS) and dampened inflammatory activity [172]. A similar study examined the effect of sodium butyrate gavage (20 mg/kg body weight/day) on male Sprague-Dawley rats consuming HFD (58% kcal fat) for 6 weeks found that butyrate supplementation decreased pro-inflammatory markers interleukin-6 (IL-6) and NF-κB and increased protein content of the inhibitor of nuclear factor kappa B, alpha (IκBα) in the liver [173]. Proteins related to fatty acid catabolism and greater insulin sensitivity, including peroxisome proliferator-activated receptor α (PPAR-α) and γ (PPAR-γ), were reduced in the HFD-fed rats; and butyrate supplementation significantly increased their expression [173]. Additionally butyrate-supplemented rats exhibited significantly increased hepatic peroxisome proliferator-activated receptor γ coactivator 1-α (PGC-1α), a transcriptional coactivator important in increasing mitochondrial biogenesis and function, expression [173]. In a murine model of autoimmune hepatitis induced by Freund’s complete adjuvant, male C57BL/6 mice receiving 300 mg/kg sodium butyrate as a daily gavage for 3 weeks exhibited reduced mRNA and protein content of NF-κB, IL-6 and TNF-α relative to the hepatitis controls [174]. Finally, Liu and colleagues administered *C. butyricum* (5 × 10^8^ CFU) intragastrically in male ICR mice for 5 days prior to acute liver injury induced via carbon tetrachloride (CCl_4_) intraperitoneal injection [175]. The pretreatment with *C. butyricum* exerted a prophylactic effect against CCl_4_ injection: ICR mice that received the bacterial strain showed improvements in markers of oxidative stress, including significantly greater nuclear factor (erythroid derived-2)-like 2 (NRF-2) protein content and elevated superoxide dismutase (SOD) and catalase enzyme activity and markers of pro-inflammation, including reduced TNF-α and increased anti-inflammatory cytokine IL-10 [175]. A summary of the effects of butyrate and its potential mechanisms of action can be found in Figure 1.

### 5.2. Skeletal Muscle

**Figure 2:**
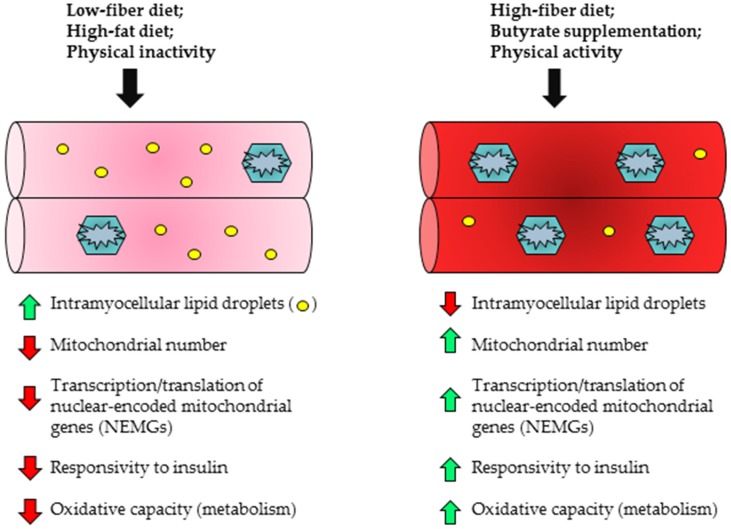
Effects of butyrate on skeletal muscle physiology.

Skeletal muscle represents one of the largest insulin responsive tissues and utilizes a significant proportion of blood glucose during metabolism [176]. In cases of obesity or T2D, glucose uptake and fatty acid oxidation become compromised, leading to increased ectopic lipid deposition in the skeletal muscle [177,178]. These changes often occur in conjunction with reduced expression of genes involved in mitochondrial biogenesis and function, thereby decreasing the capacity for oxidative metabolism [179,180]. Chronic overfeeding has also been associated with impaired substrate transitioning and generation of reactive oxygen species (ROS) by the mitochondria, which may ultimately interfere with insulin signaling pathways [166,178,181]. In contrast to these deleterious effects, increased expression of PPAR-α has been associated with greater fatty acid oxidation capacity in skeletal muscle [182,183]. Moreover, adenoviral-mediated PGC-1α overexpression in culture human skeletal muscle myotubes was observed to increase fatty acid oxidation, elevate mitochondrial DNA (mtDNA) content by approximately 72% and modestly reduce lipid deposition [184]. The beneficial effects of butyrate supplementation on skeletal muscle have been demonstrated in both prevention and treatment-focused interventions (Figure 1 and Figure 2). For example, Hong and colleagues examined the effect of oral sodium butyrate administration in C57BL/6 mice that had already been administered HFD (45% kcal fat) for 8 weeks [18]. Half of the HFD-fed mice then received daily gavages of 80 mg sodium butyrate for 10 consecutive days, while control HFD mice received vehicle. The mice that received the sodium butyrate supplementation exhibited decreased serum insulin, leptin and fasting glucose concentrations [18]. Total body weight, liver weight and epididymal fat pad weight also decreased in butyrate-supplemented mice relative to the HFD controls [18]. Although butyrate supplementation did not alter protein expression of Ffar 3 or 4 in gastrocnemius muscle, ChIP assays revealed elevated acetylation at the gene promoter regions of adiponectin receptors 1 and 2 (*Adipor1*/*Adipor2*) and uncoupling proteins 2 and 3 (*Ucp2*/*Ucp3*) in butyrate-supplemented mice [18]. Finally, phosphorylated AMPK content was significantly elevated in butyrate-supplemented mice relative to HFD controls [18]. With regard to preventative benefits of butyrate administration, Gao et al. observed greater weight maintenance and prevention of weight gain in C57BL/6J mice that received sodium butyrate (5% wt/wt) with concurrent HFD (58% kcal fat) feeding as compared to HFD-feeding alone; the butyrate intervention also reduced adiposity, maintained muscle mass and resulted in greater conversion of type II (glycolytic) muscle fibers to type I (oxidative) fibers [6]. Western blotting also revealed elevated PGC-1α, type I myosin heavy chain and phosphorylated AMPK protein content in butyrate-treated mice [6]. Sodium butyrate supplementation has also been shown to epigenetically regulate nuclear-encoded mitochondrial gene expression, increasing the expression of genes necessary for beneficial mitochondrial adaptations, while also preventing HFD-induced incomplete β-oxidation in skeletal muscle [7]. For example, in mice fed a HFD supplemented with 5% wt/wt sodium butyrate, there was a significant increase in *Pgc-1α* gene expression that was associated with prevention of obesity and insulin resistance as well as improved mitochondrial function and a higher percentage of type 1 oxidative fibers in skeletal muscle [7]. Thus butyrate may prevent obesity and insulin resistance partially by acting on the skeletal muscle to improve its oxidative capacity.

### 5.3. Adipose Tissue

The anti-obesogenic and diabetic effects of butyrate supplementation may be partially attributed to its metabolic effects on adipose tissue (Figure 1). Butyrate treatment in vitro has been shown to increase lipolysis in 3T3-L1 cells. This increase may be due to its HDACi activity, as the HDACi trichostatin A similarly increases lipolysis in 3T3-L1 cells [185]. Although the authors conclude that butyrate-induced lipolysis may induce insulin resistance due to increased plasma FFA levels, investigators have shown that butyrate increases fatty acid oxidation in peripheral tissues, such as muscle and adipose tissue [6,7]. Thus, it is possible that butyrate-induced lipolysis improves insulin sensitivity when coupled with butyrate-induced increases in oxidation of fatty acids. In fact, a recent study by Li et al., 2017, shows that oral butyrate supplementation increases fatty acid oxidation in brown adipose tissue in addition to preventing diet-induced obesity and insulin resistance [186]. The authors also noted that increased fatty acid oxidation was due to increased sympathetic nervous system outflow. Because butyrate has also been shown to increase B-adrenergic receptor profiles in adipocytes, which occurs via its HDACi activity [187,188], a similar mechanism for upregulating fatty acid oxidation may occur in white adipose tissue. The HDACi activity of butyrate has also been associated with its ability to prevent adipose tissue inflammation, a contributing factor to insulin resistance during obesity [189]. Butyrate also causes browning of white adipose tissue, with decreased adipocyte size and increased number of multilocular cells [6]. Consistent with these data butyrate has been found to induce adipocyte differentiation in preadipocyte 3T3-L1 cells and this butyrate-induced differentiation occurs in conjunction with increased FFAR2 expression [190]. Adipocyte differentiation is implicated in improving insulin sensitivity and impairment with insulin resistance [191,192]. Thus, the adipogenic promoting properties of butyrate may play a role in its known beneficial insulin-sensitizing effects. In contrast to its lipolytic effects butyrate’s adipogenic effects occur concomitant to increases in FFAR2 expression, suggesting that these effects occur through GPCR signaling rather than its HDACi activity [190]. As previously discussed butyrate also acts through the FFAR3 receptor and in adipose tissue FFAR3 activation by SCFA increases leptin production [193]. Butyrate-induced increased levels of leptin may be important in preventing obesity, as leptin acts within the hypothalamus to reduce food consumption and increase energy expenditure. In fact, others have shown that oral butyrate supplementation depresses orexigenic neuronal activity and decreases food intake [186]; however, others have shown no effect of dietary supplementation of butyrate on food intake [6,7].

## 6. Butyrate and Clinical Studies

Although in vitro and in vivo animal studies support the ability of butyrate supplementation to prevent colon cancer, obesity and insulin resistance, few studies in human participants have been conducted to date to corroborate the beneficial results of butyrate supplementation. Human studies have primarily emphasized changes in gut microbial diversity (e.g., Bacteroidetes-to-Firmicutes ratio) following dietary intake of resistant starches or other fermentable carbohydrates [194,195,196], some of which have been identified as butyrogenic, rather than dietary supplementation with butyrate itself. While several clinical interventions have shown beneficial effects of altering microbiota on reduced body weight and fat mass, as well as increased insulin sensitivity (Table 1) [48,197,198,199]; at least one clinical trial has failed to demonstrate that microbial changes exert beneficial effects on body weight or insulin sensitivity in overweight/obese individuals (Table 1) [200]. Of note, this study also showed no effect on serum butyrate levels [200]; thus, the lack of beneficial effects may be due to the inability of the intervention to change butyrate levels rather than an absent effect of butyrate. Another recent study has shown that 7 days of antibiotic treatment, which alters the gut microbiota to decrease butyrate-producing bacteria and plasma and fecal levels of butyrate, has no effect on insulin sensitivity or energy expenditure in overweight individuals [201]. As the present review focuses on interventions that increase butyrate and few studies have looked at the effects of decreasing butyrate, it is unclear whether one would expect a decrease in butyrate to have any effects on body weight, insulin sensitivity or metabolic parameters or the acute (7 days) expected effects of decreasing butyrate. Thus, further clinical research is warranted. Additionally, increased consumption of dietary fiber has been linked to decreased adiposity and body weight in clinical studies; however, the role of butyrate itself and the mechanisms involved are unclear. Although several putative mechanisms have been proposed in animal models including the release of anorectic hormones and increased degradation of fatty acid species, continued research with human participants is necessary to more fully address these possibilities [202,203].

## 7. Conclusions

Butyrate, a SCFA essential for the proper growth and function of the gastrointestinal epithelium, is primarily derived from the fermentation of dietary fibers and resistant starches by the colonic microbiota. Beyond its HDACi action butyrate serves as a fuel source for enterocytes and colonocytes, facilitates apoptosis of colonic cancer cells and reduces gut inflammation. Although additional work is needed to determine the levels of butyrate reaching and affecting peripheral tissues and a mechanism of action within these tissues, the literature has shown that butyrate supplementation has the ability to prevent obesity and insulin resistance through its actions in peripheral tissues. Mainly butyrate supplementation acts to decrease ectopic lipid deposition and inflammation and produces mitochondrial adaptations that increase β-oxidation of fatty acids. Given the beneficial effects of butyrate found in animal and clinical studies, dietary strategies that can increases butyrate levels may serve as likely treats to mitigate cancer, obesity and T2D. Thus, additional translational and clinical studies in these areas are warranted.

## Figures and Tables

**Table 1:** Effects of butyrate, dietary fiber, or microbial transplantation treatment on body weight, body composition, inflammation, satiety hormones and/or insulin sensitivity in human participants. Upward arrows (↑) indicate an increase and downward arrows (↓) indicate a decrease in respective measured outcomes.

**Acute Study**	**Short Chain Fatty Acid(s) or Dietary Fiber Composition**	**Control Group**	**Route of Administration**	**Duration of Treatment**	**Findings in Treatment Group Relative to the Control Group**	**Findings in Treatment Group Relative to Themselves (Longitudinal)**
Nilsson et al., (2008) [199]	White wheat flour bread supplemented with barley fiber or resistant starch cereal-based meals (50 g available starch)	Unsupplemented white wheat flour bread (50 g available starch)	Oral—dietary supplement	2 meals	↑ glucose tolerance following a meal, GLP-1 and satiety; ↓ serum free fatty acids in men and women	
Vrieze et al., (2012) [204]	Microbial transplantation from lean donors	Microbial transplantation from own collected feces	Bowel lavage	Single intervention—measurements made 6 weeks after infusion		↑ peripheral insulin sensitivity; no effect on resting energy expenditure in obese males with metabolic syndrome
**Chronic Study**	**Short Chain Fatty Acid(s) or Dietary Fiber Composition**	**Control Group**	**Route of Administration**	**Duration of Treatment**	**Findings in Treatment Group relative to the Control Group**	**Findings in Treatment Group Relative to Themselves (Longitudinal)**
Dewulf et al., (2013) [205]	Oligofructose (16 g/day)	Dextrin maltose (16 g/day)	Oral—dietary supplement	2 weeks	↓ postprandial glucose AUC; ↑ PYY and GLP-1 in healthy men and women	
Gower et al., (2016) [49]	Resistant starch (30 g/day)	Waxy Corn Starch matched based on digestible starch in treatment group	Oral—dietary supplement	4 weeks	↑ insulin sensitivity in insulin resistant women; no effect in insulin sensitive women	
Parnell et al., (2009) [198]	Oligofructose (21 g/day)	Maltodextrin (21 g/day)	Oral—dietary supplement	12 weeks	↓ body weight, energy intake, fat mass and trunk fat, serum glucose and insulin and active ghrelin; ↑ PYY; no effect on GLP-1 in overweight/obese men and women	
Pedersen et al., (2013) [206]	Oligofructose (15–55 g/day)	none	Oral—dietary supplement	5 weeks		↑ PYY and satiety; no effects on energy intake, glucose, insulin, or GLP-1 in men and women
Robertson et al., (2005) [48]	Resistant starch (30 g/day) + 20 g/day rapidly digestible starch	Rapidly digestible starch (Amioca; 20 g/day)	Oral—dietary supplement	4 weeks	↑ insulin sensitivity during meal tolerance test; ↑ glucose uptake by adipose tissue	
Scheppach et al., (1997) [207]	Sodium butyrate enema (100 mmol/L) or SCFA mixture (butyrate = 40 mmol/L	isotonic saline	Rectal	8 weeks		↓ polymorphonuclear leukocytes in lamina propria; ↓ upper intestinal crypt proliferation in individuals with active distal ulcerative colitis
	acetate = 60 mmol/L		(twice daily)			
	propionate = 30 mmol/L)					
Vulevic et al., (2013) [208]	Galactooligosaccharide (5.5 g/day)	Maltodextrin (5.5 g/day)	Oral—dietary supplement	12 weeks	↓ fasting insulin, triglycerides, total cholesterol and C-reactive protein in overweight/obese men and women	
Wolever et al., (2002) [209]	High fiber cereal (10–15% energy)	Low fiber cereal (10–15% energy)	Oral	6 months		No effect on body weight, serum triacylglycerols or total cholesterol; nonsignificant ↓ HDL cholesterol in type 2 diabetic men and women

GLP-1, glucagon-like peptide-1; PYY, peptide YY; HDL, high density lipoprotein.
